# Protocol for the generation and differentiation of thymic epithelial organoids from adult mouse thymus tissue

**DOI:** 10.1016/j.xpro.2025.103883

**Published:** 2025-06-10

**Authors:** Sam Willemsen, Ni Luh Wisma Eka Yanti, Johan H. van Es, Hans Clevers, Sangho Lim

**Affiliations:** 1 Hubrecht Institute, Royal Netherlands Academy of Arts and Sciences (KNAW) and University Medical Center Utrecht, 3584 CT Utrecht, the Netherlands; 2 Oncode Institute, 3521 AL Utrecht, the Netherlands

**Keywords:** Cell Biology, Cell culture, Molecular Biology, Organoids

## Abstract

Here, we present a protocol for generating long-term expandable thymic epithelial cell (TEC) organoids from adult mouse thymus. We describe the steps for tissue isolation, dissociation, 3D culture matrix embedding, organoid maintenance, and passaging. We then detail procedures for TEC differentiation into cortical and medullary TEC lineages using defined growth factors, and techniques for analyzing organoids, including flow cytometry and qPCR. This protocol provides a valuable tool for studying TEC development and function.

For complete details on the use and execution of this protocol, please refer to Lim et al.^1^

## Highlights

• Steps for thymus tissue excision from mice and initial plating for organoids
• Procedures for TEC organoid freezing, thawing, and passaging
• Procedures for TEC organoid differentiation into mTEC and cTEC lineages
• Guidance on TEC organoid characterization by qPCR and flow cytometry

## Abstract (editor highlights)

Publisher’s note: Undertaking any experimental protocol requires adherence to local institutional guidelines for laboratory safety and ethics.

## Graphical abstract

**Figure:**
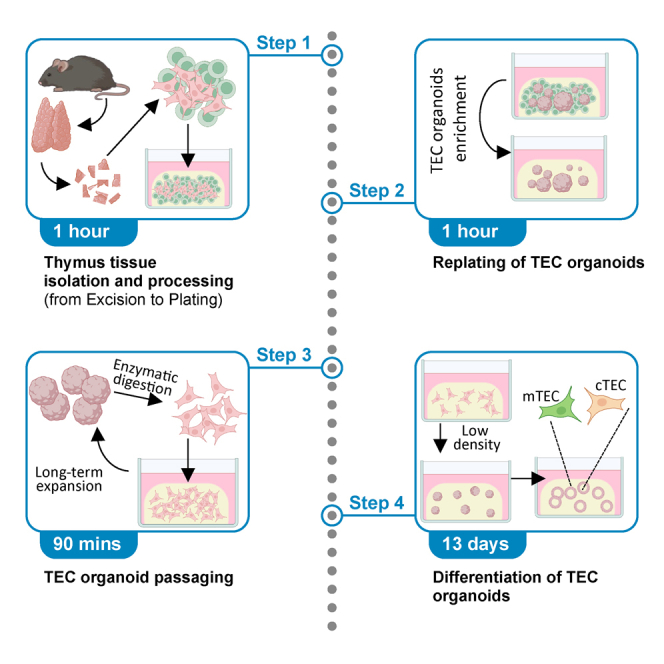

## Before you begin

Thymic epithelial cells (TECs) play a crucial role in T cell development and immune system homeostasis by providing essential signals for thymocyte maturation and selection.^2^^,^^3^ The ability to generate thymic epithelial cell organoids from adult mouse thymus tissue offers a powerful *in vitro* system to study thymic epithelial biology, immune cell interactions, and potential regenerative approaches for thymus dysfunction. Unlike traditional 2D culture systems, three-dimensional TEC organoids provide a long-term expandable model with the potential to support studies on thymic development, aging, and related disorders.

To generate thymic epithelial cell (TEC) organoids from adult mouse thymus tissue, proper preparation and handling of tissue samples are critical. This protocol requires the use of adult C57BL6 mouse thymus tissue (6–12 weeks old) and emphasizes immediate processing after isolation to ensure cell viability. Prepare all reagents, including defined growth factor-enriched medium and extracellular matrix (ECM), before starting the protocol. Use aseptic techniques throughout the process to prevent contamination during tissue dissociation, 3D embedding, and organoid culture. Additionally, ensure access to equipment and materials for downstream analyses, such as flow cytometry, qPCR, and immunostaining, which are used to assess organoid differentiation and functionality.

Researchers should obtain appropriate institutional ethical approvals before conducting experiments involving animal tissue. Adherence to institutional guidelines for animal care and handling is essential to ensure compliance with ethical research standards.

### Institutional permissions

All procedures described in this protocol were approved by the institutional animal ethics committee of the Royal Netherlands Academy of Arts and Science (KNAW) under project license AVD8010020151. Researchers intending to perform this protocol must obtain approval from their respective institutional ethics committees before initiating experiments.

### Preparation of TEC organoid media

**Timing: 1 h**

1. Preparation of chemical and growth factor stocks.

**Table:** 

Reagent	Amount	Solvent	Volume	Stock concentration
N-Acetyl-L-cysteine	4 g	Milli-Q Water	49 mL	500 mM
Nicotinamide	12.2 g	PBS	100 mL	1 M
EGF	1 mg	0.1% BSA in PBS	2 mL	500 μg/mL
A83-01	50 mg	DMSO	23.7 mL	5 mM
FGF7	100 μg	0.1% BSA in PBS	1 mL	100 μg/mL
FGF10	100 μg	0.1% BSA in PBS	1 mL	100 μg/mL
sRANK-ligand	100 μg	0.1% BSA in PBS	1 mL	100 μg/mL
Retinoic acid	10 mg	DMSO	3.33 mL	10 mM
Y-27632 dihydrochloride	100 mg	Milli-Q Water	31.2 mL	10 mM

Store at −80°C for up to 12 months. Avoid repeated freeze/thaw cycles.

2. AdDF+++ medium.

***Note:*** The medium can be used to store isolated thymus tissues temporarily, to chop the tissues using scissors, and to prepare expansion medium, 1^st^ differentiation medium (DM), or 2^nd^ differentiation medium (DM+RR).

**Table:** AdDF+++ medium

Reagent	Final concentration	Amount
Advanced DMEM/F12	N/A	485 mL
Penicillin-Streptomycin (10,000 U/mL)	100 U/mL	5 mL
HEPES (1 M)	10 mM	5 mL
GlutaMAX (200 mM)	2 mM	5 mL
**Total**	**N/A**	**500 mL**

Store at 4°C for up to 1 month.

3. Expansion medium.

**Table:** Expansion medium

Reagent	Final concentration	Amount
AdDF+++	N/A	477 mL
Rspo3-Fc Fusion Protein Conditioned Medium (100X)	1X	5 mL
Noggin-Fc Fusion Protein Conditioned Medium (100X)	1X	5 mL
WNT Surrogate-Fc Fusion Protein (0.5 μM)	0.5 nM	500 μL
B-27 Supplement without Vitamin A (50X)	1X	10 mL
EGF (500 μg/mL)	50 ng/mL	50 μL
FGF7 (100 μg/mL)	50 ng/mL	250 μL
FGF10 (100 μg/mL)	50 ng/mL	250 μL
A83-01 (5 mM)	500 nM	50 μL
Nicotinamide (1 M)	2.5 mM	1.25 mL
N-acetylcysteine (500 mM)	625 μM	625 μL
**Total**	**N/A**	**500 mL**

Store at 4°C for up to 1 month.

***Optional:*** To prevent microbial contamination, Primocin can be freshly added to the medium immediately prior to use to achieve a final concentration of 50 μg/mL.

***Note:*** The concentration of WNT surrogate-Fc fusion proteins varies depending on the lot number. Therefore, pre-dilution should be performed according to the manufacturer’s datasheet, and the optimal final concentration should be empirically determined. Typically, 0.5 nM is effective for promoting organoid expansion.

4. Expansion medium with Y-27632 dihydrochloride (Rho-kinase inhibitor).

***Note:*** For the initial plating of dissociated cells from thymus tissues, expansion medium containing Y-27632 dihydrochloride should be used. For passaging established organoids, expansion medium with Y-27632 dihydrochloride should be used for the first 2 to 3 days.

**Table:** Expansion medium containing Y-27632 dihydrochloride (Rho-kinase inhibitor)

Reagent	Final concentration	Amount
Expansion medium	N/A	49.95 mL
Y-27632 dihydrochloride (10 mM)	10 μM	50 μL
**Total**	**N/A**	**50 mL**

Prepare immediately before use.

5. 1^st^ Differentiation medium (DM).

**Table:** 1^st^ differentiation medium (DM)

Reagent	Final concentration	Amount
AdDF+++	-	98 mL
B-27 Supplement (50X)	1X	2 mL
**Total**	**N/A**	**100 mL**

Store at 4°C for up to 1 month.

***Note:*** In the expansion medium, B-27 Supplement without vitamin A is used, whereas in the differentiation medium (both DM and DM+RR), the standard B-27 Supplement containing vitamin A is employed.

6. 2^nd^ Differentiation medium (DM+RR).

**Table:** 2^nd^ differentiation medium (DM+RR)

Reagent	Final concentration	Amount
AdDF+++	–	97.79 mL
B-27 Supplement (50X)	1X	2 mL
sRANK-ligand (100 μg/mL)	200 ng/mL	200 μL
Retinoic acid (10 mM)	1 μM	10 μL
**Total**	**N/A**	**100 mL**

Store at 4°C for up to 1 month.

7. Preparation of BME Cultrex Basement Membrane Extract (BME), Growth Factor Reduced, Type 2, should be thawed at 4°C for at least 16 h in advance. Once removed from the refrigerator, it should always be kept on ice during use.

***Note:*** After thawing, BME can be stored at 4°C for up to one month. While in use, it should always be kept on ice.

## Key resources table

**Table:** 

REAGENT or RESOURCE	SOURCE	IDENTIFIER
**Biological samples**		

C57BL/6 mice (female used; male also possible)	Hubrecht Institute	N/A

**Chemicals, peptides, and recombinant proteins**		

Cultrex basement membrane extract (BME), growth factor reduced, type 2	R&D Systems, Bio-Techne	3533-005-02
DPBS, no calcium, no magnesium	Thermo Fisher Scientific	14190144
Bovine serum albumin	Sigma-Aldrich	A7888
Advanced DMEM-F12	Thermo Fisher Scientific	12634–010
Penicillin-Streptomycin	Thermo Fisher Scientific	15140122
Primocin	InvivoGen	ant-pm-2
HEPES	Thermo Fisher Scientific	15630080
GlutaMax	Thermo Fisher Scientific	35050061
B-27 Supplement without Vitamin A	Thermo Fisher Scientific	12587010
Nicotinamide	Sigma-Aldrich	N0636
N-Acetyl-L-cysteine	Sigma-Aldrich	A9165
EGF	Thermo Fisher Scientific	AF-100-15
A83-01 (ALK4/5/7 inhibitor)	Tocris	2939
FGF10	Thermo Fisher Scientific	100-26
FGF7	Thermo Fisher Scientific	100-19
WNT Surrogate-Fc fusion protein	IPA therapeutics	N001 - 100 μg
Noggin-Fc fusion protein conditioned medium	IPA therapeutics	N002 - 200 ml
Rspo3-Fc fusion protein conditioned medium	IPA therapeutics	R001 - 500 ml
Y-27632 dihydrochloride (Rho-kinase inhibitor)	AbMole	M1817
B-27 Supplement	Thermo Fisher Scientific	17504044
sRANK-ligand	Thermo Fisher Scientific	315-11C
Retinoic acid	Sigma-Aldrich	R2625
TrypLE Express Enzyme (1X), phenol red	Thermo Fisher Scientific	12605010
Cellbanker I	AMSBIO	11910

**Other**		

6-well suspension culture plate	Greiner Bio-One	657185
15 mL conical tube	Greiner Bio-One	188271
100 μm cell strainer (for 15 mL conical tube)	Greiner Bio-One	542100
40 μm cell strainer (for 15 mL conical tube)	Greiner Bio-One	542140
CELLSTAR dish ∅10 cm	Greiner Bio-One	664160

## Step-by-step method details

### Isolation and processing of thymic tissue

**Timing: 1h**

This step involves the euthanasia of mice, followed by the isolation, cleaning, and mechanical dissociation of thymic tissue. The excision of murine thymus is based on a previously published protocol.^4^ The thymus is finely minced without enzymatic digestion, preparing the sample for the subsequent steps.

1. Preparation steps required before tissue collection.
  a. Prepare sterile tools and materials, including scissors, forceps, gauze pads, and 2 mL tubes.
  b. Pre-cool the centrifuge to 4°C.
  c. Place BME on ice to keep it cold.
2. Euthanize five wild-type C57BL/6 mice, 6 to 12 weeks old.

***Note:*** Using less number of mice is also possible, however, five mice helps to ensure a sufficient number of isolated cells to generate organoids, especially when following this protocol for the first time.

***Note:*** Euthanasia should be performed in accordance with the approved protocol. However, cervical dislocation is not recommended, as it may cause intrathoracic hemorrhage in certain cases, which can result in clotted blood adhering to the thymus. In this protocol, the animals were euthanized using carbon dioxide (CO_2_) inhalation, in accordance with institutional and ethical guidelines.

3. Isolate thymus while minimizing blood contamination.
  a. Make a midline incision through the skin using surgical scissors, from above the urethral opening to the lower jaw.
  b. Extend the incision downward on both sides toward the knees, creating an inverted “Y” shape.
  c. Gently pull back the loosened skin and secure it to the dissection mat with pins.
  d. Puncture the diaphragm near the xiphoid process and carefully cut along both sides of the ribcage up to the clavicle.
  e. Using forceps, lift the ribs and pin them to the dissection mat to expose the thoracic cavity.
  f. The thymus, appearing as two thin and whitish lobes, positioned over the heart, can be observed under the ribs (Figure 1A).

**Figure 1:**
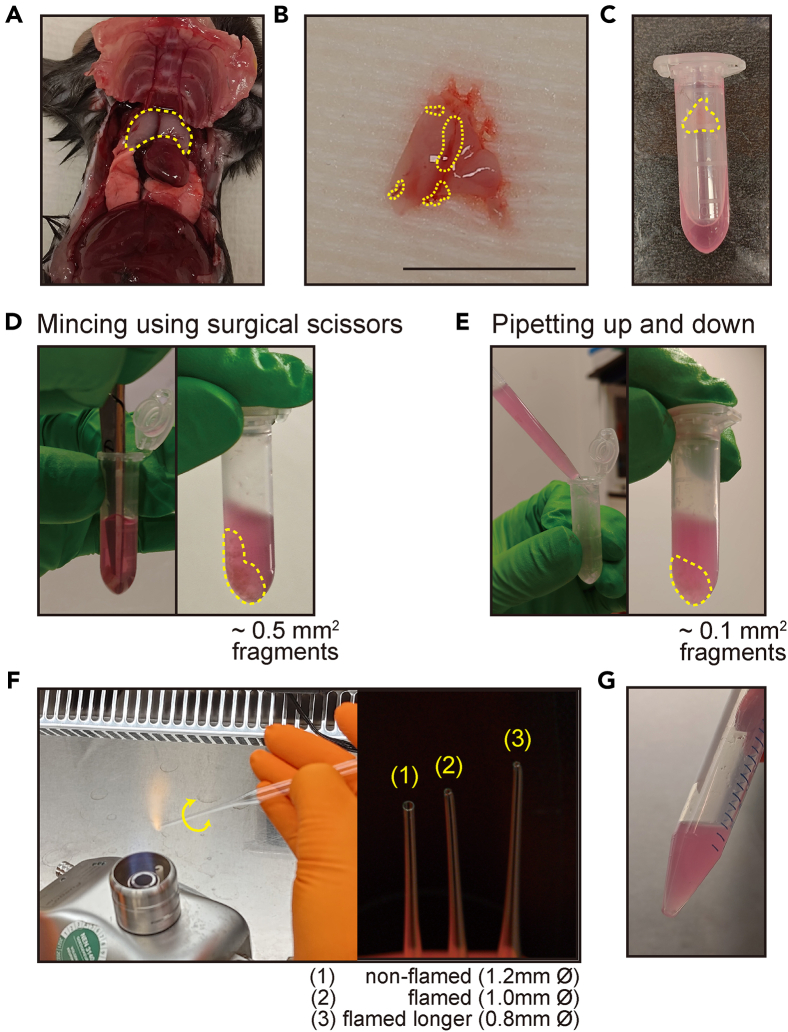
Tissue isolation and mechanical dissociation for TEC organoid generation (A) Dissection of the adult mouse thymus (yellow circle with dashed line). (B) Isolated thymus tissue before processing. Scale bar = 1 cm. (C) Tissue transferred into a tube containing AdDF+++ (yellow arrow). (D) Mincing of thymus tissue using surgical scissors to generate approximately 0.5 mm^2^ fragments. (E) Further dissociation by pipetting up and down to produce smaller tissue fragments. (F) Glass pipette modifications for dissociation: (1) non-flamed (wide opening), (2) flamed (moderate opening), (3) flamed longer (narrow opening). (G) Representative image after the dissociation process, showing no visible tissue fragments, ready for further processing.
  g. Carefully detach the surrounding connective tissue and extract both thymic lobes using tweezer.
    ***Note:*** The following steps should be performed under aseptic conditions to prevent contamination. Use sterile tools and work in a biosafety cabinet.
  h. Place the excised thymus on a piece of sterile gauze moistened with sterile PBS (Figure 1B).
  i. Using sterile surgical scissors and tweezer, remove as much visible connective tissue and surrounding fat (Figure 1B) as possible.
  j. Immerse the thymus in a 10 cm Petri dish containing 10 mL of AdDF+++ medium, repeatedly dipping it in and out to thoroughly rinse the surface.
  k. Transfer the excised thymus into a 2.0 mL microcentrifuge tube containing 1 mL of AdDF+++ medium (Figure 1C).
4. Mince the thymus into pieces of 0.5 mm^2^ using scissors (Figure 1D).

**CRITICAL:** The mincing process is performed inside the microcentrifuge tube. Cut the thymus into the smallest possible fragments using surgical scissors. Since this protocol does not involve enzyme digestion, mincing the tissue as finely as possible is crucial.

5. Pipette the tissue pieces up and down repeatedly with a 1000 μL pipette tip to disrupt the tissue fragments until they are homogenous enough to be pipetted up and down smoothly (Figure 1E).

***Note:*** Each thymus is processed individually in a separate microcentrifuge tube, following the steps above. This means that steps 2–4 should be repeated for all five thymi. After this point, the samples will be combined in the following steps.

6. Pool all finely minced thymus tissue fragments in a 15 mL conical tube.
7. Pellet the tissue fragments by centrifuging at 300 x g for 3 min at 4°C.
8. Add 2 mL of AdDF+++ medium and resuspend the tissue fragments using a flamed (narrowed) glass Pasteur pipette (Figure 1F) until they can be pipetted up and down smoothly (Figure 1G).

***Note:*** To prepare a flame-narrowed Pasteur pipette, hold the tip of the pipette in the flame for a few seconds while rotating the glass pipette (Figure 1F). If the opening is too narrow from the start, tissue fragments may become lodged, making pipetting up and down difficult or impossible. To avoid this, gradually narrow the pipette tip in two steps, ensuring that pipetting remains feasible throughout the process. To minimize adhesion of tissue fragments to the glass pipette, first pipette 1% BSA in PBS up and down several times to pre-wet the inner surface before use. If only a minimal amount of tissue adheres to the pipette, you may proceed to the next step without concern.

9. Pass the tissue pieces through a 100 μm cell strainer and collect them in a new 15 mL conical tube.
10. Rinse the cell strainer with an additional 10 mL of AdDF+++ to collect any remaining cells into the same 15 mL conical tube.
11. Pellet the sample by centrifuging at 500 x g for 5 min at 4°C. Carefully aspirate the supernatant.
12. Resuspend in 10 mL of AdDF+++ and repeat step 10.
13. Carefully aspirate the supernatant.

### Generation of TEC organoid cultures

**Timing: 1h, including approximately 30 min of plate incubation time**

This step enables the formation of 3D TEC organoids within BME droplets. A mixture of epithelial cells and other cell types isolated from tissue fragments is plated in BME. Regular medium changes with expansion medium favor epithelial cell growth and organoid development, gradually depleting non-epithelial cells. By day 14, mature epithelial structures should be visible and ready for further processing.

**Figure 2:**
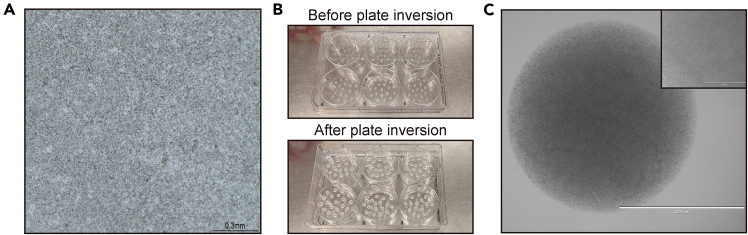
Initial plating for TEC organoid culture (A) Representative bright-field image showing the proper level of thymus tissue dissociation before mixing with BME. Tissue fragments should be sufficiently dissociated to prevent large aggregates. Scale bar = 0.3 mm. (B) Plate inversion technique to reduce attachment of the cells to the plate surface. (C) Bright-field image of thymus-derived cells immediately after plating. At this stage, all dissociated cells from the thymus, including epithelial and non-epithelial populations, are present within the BME droplets, resulting in high initial density. Scale bar = 2000 μm.

***Note:*** BME is viscous, making direct resuspension challenging. Pre-mixing the pellet with expansion medium ensures a more uniform suspension before adding BME.

14. Resuspend the pellet in 600 μL of Expansion medium (Figure 2A).
15. Add 3 mL of BME and mix carefully by pipetting. Resuspend gently to prevent air bubble formation and ensure thorough mixing.

16. Plate the BME-cell mixture into wells of a 6-well plate to form small droplets of approximately 10 μL each.

***Note:*** Ensure that each well contains a total of 200 μL of the BME-cell mixture suspension. This should result in approximately 20 droplets per well. Based on previous experiments, pooling cells from five mouse thymi typically yields a sufficient number (approximately 2 to 4 X 10^8^) of cells to be plated across three full 6-well plates.

***Note:*** To prevent the BME-cell mix droplets from spreading out and becoming too flat, it is recommended to use a suspension plate with a hydrophobic surface. If using a standard cell culture plate, it should be pre-warmed at 37°C for at least 24 h before use, as insufficient pre-warming may result in poor dome formation. However, this protocol exclusively utilizes suspension plates.

17. Gently invert the plate and leave it at 22°C for approximately 3 min (Figure 2B). Then, place the plate in a 37°C, 5% CO_2_ incubator.

**CRITICAL:** The plate should be inverted to prevent cells from settling at the bottom due to gravity. If various cell types at this step, including epithelial cells, adhere to the surface and grow, they may form a 2D culture instead of a 3D organoid (see problem 5). Therefore, the plate should be kept inverted during this step and throughout the incubation period until the BME is fully solidified.

18. Incubate for 30 min in the incubator to allow the BME to solidify.
19. Pre-warm the expansion medium containing Y-27632 dihydrochloride during step 18.
20. Add 2.5 mL of expansion medium containing Y-27632 dihydrochloride. Using a 10 mL serological pipette, gently dispense the medium along the side wall of the well. Avoid pipetting directly onto the BME droplets to prevent disturbance.
21. Observe the cells using a bright-field microscope to confirm that they are evenly distributed in the BME dome at high density (Figure 2C).
  ***Note:*** At this point, the droplets contain all the cells from the thymus, resulting in a highly dense cell population.
22. Refresh the expansion medium containing Y-27632 dihydrochloride every 2 to 3 days. At each medium change, completely remove the old medium and replace it with fresh medium.

**Figure 3:**
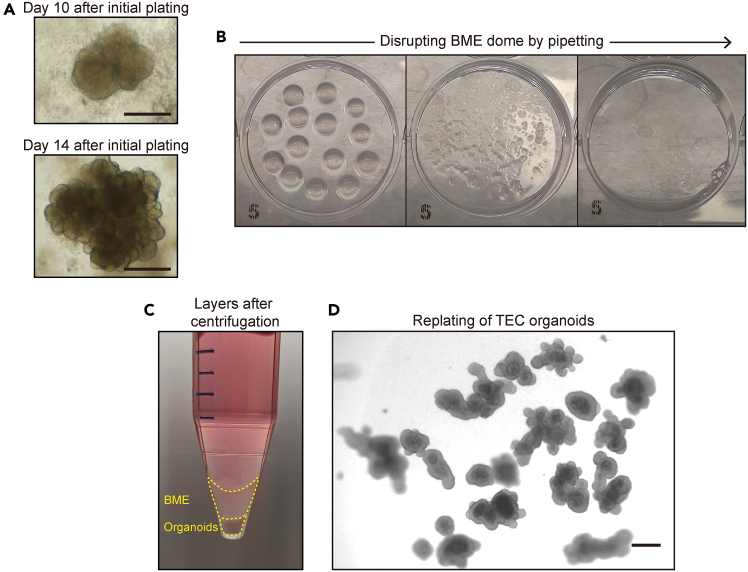
TEC organoid growth and re-plating process (A) Bright-field images of TEC organoids at day 10 (top) and day 14 (bottom) after initial plating, showing organoid growth and structural expansion. Scale bar = 100 μm. (B) Representative images showing BME disruption by pipetting. (C) Representative image showing organoids pellet, BME layer, and supernatant after centrifugation. (D) Re-plating of TEC organoids after 14 days of culture. Organoids are collected and redistributed to fresh BME droplets for continued expansion. Scale bar = 100 μm.

***Note:*** By day 10 to 14, 3D organoids should become visible (Figure 3A). However, due to the high cell density within the BME droplets, visualization may still be challenging. Adjust the microscope settings to increase the amount of light, improving penetration through the BME droplet for better observation. If organoids are not generated, refer to troubleshooting, problem 1.

**CRITICAL:** If 3D organoid formation is observed by day 14, all wells from the three 6-well plates should be collected. At this stage, non-organoid cells, including dead single-cell suspensions, will be largely washed out.

### Replating of TEC organoids for initial establishment

**Timing: 1h, including approximately 30 min of plate incubation time**

In this step, only the well-formed 3D TEC organoids are collected, enriching for epithelial cells, while most non-viable cells, including thymocytes and other cell types, are washed away. The organoids from three 6-well plates are pooled into a single 6-well plate, consolidating the culture. These enriched organoids are then used for the next step.

23. On day 14 from the initial plating, collect all plated samples by transferring the contents of each 6-well plate into a separate 15 mL conical tube.
  a. Carefully remove 1 mL of culture medium from each well and set it aside, ensuring minimal disturbance to the BME droplets.
    ***Note:*** Some 3D TEC organoids may adhere to the bottom of the plate during disrupting BME droplets by pipetting. To minimize organoid loss, set aside 1 mL of medium before collecting all BME droplets. After collection, use the reserved 1 mL of medium to rinse the well and recover any remaining organoids.
  b. Using a 1000 μL pipette, gently pipette up and down with the remaining culture medium in the well to disrupt the BME droplets (Figure 3B), helping to resuspend the organoids and minimize organoid loss.
  c. Transfer the entire contents of each well into a 15 mL conical tube, pooling all wells from the same plate into a single tube.
  d. After collection, use the set-aside 1 mL of medium to rinse the well and recover any remaining organoids.
  e. Repeat this process for the remaining two 6-well plates, collecting organoids into separate 15 mL conical tubes.
24. Centrifuge the three collected 15 mL conical tubes at 300 x g for 3 min at 4°C to allow larger organoids to settle at the bottom. Carefully remove as much of the supernatant as possible without disturbing the pellet.
25. Resuspend the pellet in 1 mL of AdDF+++ per tube by pipetting, then add 9 mL of ice-cold AdDF+++. Centrifuge again at 300 x g for 3 min at 4°C. Carefully remove as much of the supernatant as possible.
26. Repeat step 22, but this time centrifuge at 500 x g for 5 min at 4°C. Carefully remove all upper layers, including any remaining transparent BME layer and the clear medium supernatant, leaving only the organoid pellet at the bottom (Figure 3C).
27. Resuspend the pellets from all three tubes in a total of 200 μL of expansion medium, combining them into a single tube.
28. Add 1 mL of BME and mix carefully by pipetting. Resuspend gently to prevent air bubble formation and ensure thorough mixing.
29. Plate the BME-cell mixture to form small droplets of approximately 10 μL each. Use a 6-well plate, ensuring that each well contains a total of 200 μL of the BME-cell mixture. This should result in approximately 20 droplets per well.
30. Gently invert the plate and leave it at 22°C for approximately 3 min. Then, place the plate in a 37°C, 5% CO_2_ incubator.
31. Incubate for 30 min to allow BME to solidify.
32. Add 2 mL of expansion medium. Using a 10 mL serological pipette, gently dispense the medium along the side wall of the well. Avoid pipetting directly onto the BME droplets to prevent disturbance.
33. Observe the replated organoids using a bright-field microscope to confirm proper distribution within the BME dome and enrichment of TEC organoids (Figure 3D).
34. Refresh the expansion medium every 2 to 3 days.

### Passaging of TEC organoids

**Timing: 70–90 min, including approximately 30 min of plate incubation time**

This step enables the expansion of TEC organoids by digesting the larger 3D structures with TrypLE, breaking them down into a single cell suspension of proliferative progenitor-like cells. These are then replated to generate new 3D TEC organoids. Through this passaging process, long-term expansion of TEC organoids can be achieved, allowing for sustained culture and broader applications in TEC biology research.

***Note:*** Mouse TEC organoid cultures can typically be passaged at a 1:6 ratio every 14 days, though this may vary depending on experimental conditions. It is recommended to start with a 1:6 ratio; however, lower split ratios may be necessary depending on the desired growth rate. After splitting, organoids originating from 200 μL of collected BME droplets will be resuspended in a final volume of 1.2 mL of BME. To minimize reagent waste, it is advisable to determine the appropriate plating volume in advance based on specific experimental requirements.

35. On day 7 after replating (step 29), disrupt the BME droplets by pipetting up and down with the culture medium in the wells.

***Note:*** If organoids adhere to the bottom of the wells, it is recommended to follow step 23a–d, to minimize organoid loss.

36. Transfer all collected material into a 15 mL conical tube. Add ice-cold AdDF+++ up to a final volume of 12 mL and mix well by inverting the tube.

***Note:*** A maximum of three wells from a 6-well plate can be pooled into a single 15 mL conical tube. If more than three wells needed to be processed, use multiple tubes accordingly.

***Note:*** During the first replating from initial organoid generation, all wells were pooled to facilitate recovery. However, during organoid passaging, handling half a plate (three wells of 6-well plate) per tube is recommended for optimal washing and minimal loss.

37. Centrifuge at 500 x g for 5 min at 4°C.
38. Carefully remove supernatant without disturbing the organoid pellet.

***Note:*** After centrifugation, three distinct layers may be visible: (1) the organoid pellet at the bottom, (2) a translucent but cloudy BME layer in the middle, and (3) the clear medium on top (Figure 3C). While TrypLE will be used in the next step for passaging, it is most efficient to remove both the supernatant and the BME layer, leaving only the organoid pellet at the bottom. If complete removal of the BME layer is not feasible, ensure that as much of it as possible is discarded to improve process efficiency.

39. Add 2 mL of TrypLE and resuspend the pellet.
40. Incubate the tube in 37°C water bath, ensuring that the organoids do not settle at the bottom. Gently swirl the tube every minute to keep the suspension homogeneous.
41. After 5 min, use a 1000 μL pipette tip to resuspend the organoids (20 times up and down) and check whether they are dissociating efficiently. If large clumps remain, incubate for an additional 3 min in the 37°C water bath, swirling the tube every minute.
42. Once the organoids have sufficiently dissociated, continue pipetting with a 1000 μL pipette tip until the medium appears cloudy.

***Note:*** If dissociation remains incomplete, attach a 20 μL pipette tip to the 1000 μL pipette tip and continue resuspending. Ensure thorough dissociation until the suspension becomes homogeneous.

**CRITICAL:** Even if some large clumps remain, TrypLE incubation in the 37°C water bath should not exceed 10 min. Over-digestion by enzymatic treatment may negatively affect organoid formation efficiency after passaging.

43. Add 2 mL of ice-cold AdDF+++ to the tube and mix well by pipetting up and down.
44. Pass the entire sample through a 40 μm cell strainer to remove undissociated clumps.

**Figure 4:**
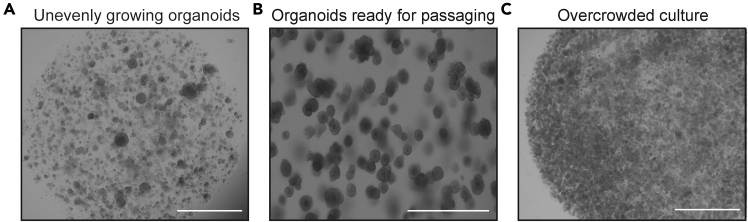
TEC organoids culture examples (A) Representative bright-field image showing unevenly growing organoids. Scale bar = 500 μm. (B) Representative bright-field image showing organoids ready for passaging. Scale bar = 200 μm. (C) Representative bright-field image showing organoids cultured under overcrowded density. Scale bar = 500 μm.

**CRITICAL:** Removing undissociated clumps by passing the sample through a 40 μm cell strainer is essential. If these clumps remain and are replated, they may start growing again, resulting in significantly larger organoids compared to others (Figure 4A). This size variation can make it difficult to maintain a uniform organoid culture, potentially complicating downstream experiments and hindering successful long-term expansion.

45. Add ice-cold AdDF+++ up to a final volume of 12 mL and mix well by inverting the tube.
46. Centrifuge at 500 x g for 5 min at 4°C. Remove the supernatant.
47. Add 1 mL of ice-cold AdDF+++ and resuspend the pellet. Then, add additional ice-cold AdDF+++ up to 10 mL and mix well by inverting the tube. Repeat step 42.
48. Resuspend the pellet in an appropriate volume of expansion medium, mix well, and then add the required volume of BME, ensuring thorough resuspension.

***Note:*** For example, if passaging was initiated from 200 μL of BME droplets (from one wells of a 6-well plate), the final plating volume should be 1.2 mL of BME droplets. In this case, resuspend the pellet in 200 μL of expansion medium, mix thoroughly, and then add 1 mL of BME, ensuring even distribution. This ratio can be used to calculate the required volume for different experimental conditions.

49. Dispense the BME-cell mixture onto the plate, forming small droplets of approximately 10 μL each. Use a 6-well plate, ensuring that each well contains a total of 200 μL of the BME-cell suspension, which should generate approximately 20 droplets per well.

***Note:*** At this stage of passaging and long-term expansion, the choice of plate format can be adjusted based on experimental scale. While a 6-well plate is commonly used, a 12-well plate or 24-well plate can also be utilized depending on the needs of the experiment. For a 12-well plate, plate 100 μL of 10 μL droplets per well. For a 24-well plate, plate 50 μL of 10 μL droplets per well.

50. Carefully invert the plate and leave it at 22°C for approximately 3 min. Then, transfer it to a 37°C, 5% CO_2_ incubator.
51. Incubate for 30 min to allow the BME to solidify.
52. Add 2 mL of expansion medium containing Y-27632 dihydrochloride into each well. Using a 10 mL serological pipette, gently dispense the medium along the well’s inner wall, ensuring minimal disturbance to the BME droplets.
53. After 3 days, replace the expansion medium containing Y-27632 dihydrochloride with fresh expansion medium (without Y-27632 dihydrochloride).
54. Continue refreshing the expansion medium every 2 to 3 days to maintain optimal culture conditions.

***Note:*** TEC organoids can be passaged approximately every 14 days (Figure 4B). However, if BME droplets become overcrowded (Figure 4C) with organoids during culture, their growth may be restricted due to limited space (see problem 3). To ensure optimal expansion, organoids can be replated after 7 days to dilute the density within BME droplets, providing sufficient space for further growth. This process follows the same step as described in “Replating of TEC Organoids” (steps 23 to 34).

***Note:*** While 14 days is a standard passaging interval, this timing can be adjusted between 10 to 20 days depending on organoid size and experimental requirements. If a longer culture period is desired, it is essential to ensure that BME droplets provide adequate space for continued organoid growth. However, to maintain high organoid viability, culturing beyond 14 days without passaging is generally not recommended.

### Freezing and thawing organoids

**Timing: 40 min for freezing (steps 51 to 57) and 60 min for thawing organoids (from step 58)**

Once TEC organoid cultures have been successfully established and passaged multiple times (two to three times), early-passage organoids can be cryopreserved for future use. Additionally, cryopreservation is not limited to early-passage organoids; long-term cultured organoids can also be frozen and revived as needed to maintain continuity in experiments. This step provides a standardized method for freezing and thawing TEC organoids, ensuring their viability and functionality upon recovery.

**Figure 5:**
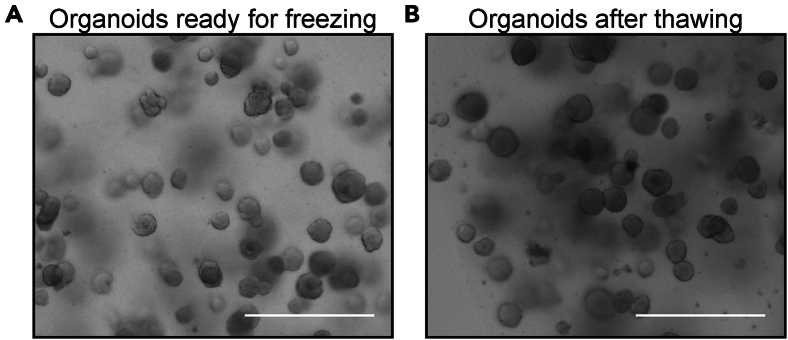
TEC organoids before freezing and after thawing (A) Representative bright-field image showing organoids ready for freezing. Scale bar = 200 μm. (B) Representative bright-field image showing organoids after thawing. Scale bar = 200 μm.

55. On day 2 or 3 post-passaging, once organoids have initiated proliferation and are no longer in a single-cell suspension state, collect BME droplets from the wells designated for freezing (Figure 5A).
  a. Carefully remove 1 mL of culture medium from each well and set it aside, ensuring minimal disturbance to the BME droplets.
  b. Using a 1000 μL pipette, gently pipette up and down with the remaining culture medium in the well to disrupt the BME droplets, allowing the existing culture medium to help resuspend the organoids and minimize organoid loss. Transfer the entire contents into a 15 mL conical tube.
  c. Use the set-aside 1 mL of culture medium to rinse each well, pipetting up and down to collect any remaining organoids, and transfer the rinse into the same 15 mL conical tube.
  d. Repeat this process for all remaining wells, pooling the collected organoids into the 15 mL conical tube.

**CRITICAL:** To ensure efficient recovery and proliferation after thawing, it is essential to freeze organoids at an early stage, specifically 2–3 days post-passaging when they are still small and actively proliferating. This timing is crucial for maximizing viability and promoting robust regrowth into larger organoids.

***Note:*** A single conical tube should contain no more than 600 μL of BME droplets, equivalent to three wells of a 6-well plate. If processing a larger volume, use multiple tubes as needed.

56. Add ice-cold AdDF+++ up to 12 mL and centrifuge at 500 x g for 5 min at 4°C.
57. Carefully remove the supernatant, ensuring that only the organoid pellet at the bottom remains. If a slightly opaque BME layer is present, remove as much as possible.
58. Resuspend the pellet in 1 mL of ice-cold AdDF+++ by pipetting, then add an additional 10 mL of ice-cold AdDF+++. Centrifuge again at 500 x g for 5 min at 4°C.
59. Remove the supernatant completely, leaving only the organoid pellet.
60. Resuspend the pellet in Cell Banker I solution, calculating the volume based on the initial collection volume. For example, organoids collected from 200 μL of BME droplets should be resuspended in 1 mL of Cell Banker I solution and preserved in 1 cryovial.
61. Transfer the resuspended organoids into cryovials and store at −80°C for up to 6 months. For longer than 6 months, store the cryovials in liquid nitrogen.
62. To thaw organoids:
  a. Prepare a container with dry ice and transfer one cryovial from −80°C storage into the container for transport.
  b. Thaw the cryovials quickly by placing them in a 37°C water bath until Cell Banker I solution is completely liquefied.
  c. While thawing, prepare a 15 mL conical tube containing 10 mL of ice-cold AdDF+++.
  d. Transfer the thawed organoid suspension from the cryovial into the 15 mL conical tube containing 10 mL of ice-cold AdDF+++.
63. Centrifuge at 500 x g for 5 min at 4°C.
64. Carefully remove the supernatant and resuspend the pellet in 1 mL of ice-cold AdDF+++ by pipetting. Then, add an additional 10 mL of ice-cold AdDF+++ and centrifuge again at 500 x g for 5 min at 4°C.
65. Remove the supernatant completely, leaving only the organoid pellet.
66. Carefully resuspend the organoid pellet in 30 μL of expansion medium, ensuring that no air bubbles form. Then, add 170 μL of BME and gently resuspend again, avoiding bubble formation.
67. Plate the BME-organoid droplets into a single well of a 6-well plate.

***Note:*** When replating thawed organoids, ensure that the same volume of BME as used prior to freezing is plated. This allows for effective recovery and ensures proper organoid growth evaluation.

68. Invert the plate and leave it at 22°C for approximately 3 min, then transfer it to 37°C, 5% CO_2_ incubator while maintaining the inverted position.
69. Incubate the plate for 30 min.
70. Carefully add 2 mL of expansion medium supplemented with Y-27632 dihydrochloride to each well. Using a 10 mL serological pipette, dispense the medium gently along the inner wall of the well to avoid disturbing the BME droplets.
71. Observe the thawed organoids using a bright-field microscope to confirm proper distribution and viability (Figure 5B).
72. After 3 days, replace the Y-27632-containing expansion medium with fresh expansion medium lacking Y-27632 dihydrochloride to support further organoid growth.
73. Refresh the expansion medium every 2 to 3 days to sustain optimal culture conditions. The organoids are now to be treated as part of the passaging cycle for continued long-term expansion.

**CRITICAL:** Organoids should be passaged at least once after thawing before use in experiments. Since not all organoids survive thawing, it is good practice to avoid using them immediately after thawing (see problem 6). After passaging, organoids will generally exhibit the same appearance as in standard culture conditions.

### Differentiation of TEC organoids

**Timing: total 8–13 days;1hfor organoid replating**

This step provides a protocol for differentiating TEC organoids cultured in expansion medium into cortical TEC (cTEC) or medullary TEC (mTEC). TECs maintained in expansion medium are highly proliferative and exhibit a progenitor-like state. However, functional TECs such as cTECs and mTECs, which play essential roles in the thymus, form a more mature yet heterogeneously differentiated cell population. Therefore, this differentiation protocol is crucial for generating these functionally relevant TEC subsets for further study.

**Figure 6:**
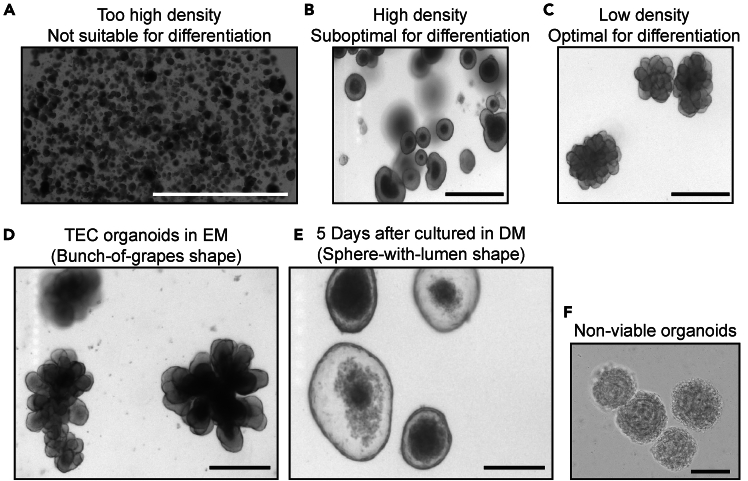
Effect of plating density on TEC organoid morphology and TEC organoid differentiation (A–C) Impact of plating density on TEC organoid growth and morphology. (A) Excessively high density results in small, tightly packed organoids, preventing proper expansion and formation of a grape-like morphology. Scale bar = 2000 μm. (B) High density is suboptimal, as organoids remain small and fail to expand fully. Scale bar = 500 μm. (C) Low density supports optimal organoid growth, allowing the development of larger structures with a distinct grape-like morphology, which is favorable for differentiation. Scale bar = 500 μm. (D and E) Morphological changes of TEC organoids in different culture conditions. (D) TEC organoids maintained in expansion medium (EM). Scale bar = 200 μm. (E) TEC organoids after 5 days in differentiation medium (DM). Scale bar = 200 μm. (F) Representative bright-field image showing non-viable organoids. Scale bar = 200 μm.

**CRITICAL:** Effective differentiation requires organoids that have reached a sufficiently large size under expansion conditions. The organoids with a diameter of approximately 150–250 μm should be transitioned to the differentiation step, as smaller organoids may not differentiate efficiently. This is achieved by replating organoids at a very low density after passaging, ensuring they have sufficient space to expand before initiating differentiation (Figures 6A–6C). If most organoids are still small but uniform in size, they are allowed to grow for an additional 1–3 days before initiating differentiation.

***Note:*** To ensure sufficient space for organoids to grow to the desired size, the replating procedure (steps 23 to 34) can be applied. To dilute the density of organoids within BME domes, one may resuspend the organoid pellet from one well of 6-well plate (200 μL BME dome) and distribute it across an entire 6-well plate. Before plating the full volume, it is recommended to first plate a single BME dome and to assess the density under a bright-field microscope. Refer Figure 4.

74. Collect the organoids into a 15 mL conical tube, following the same method used for replating and passaging, ensuring minimal disruption to preserve their structure and viability. Use one 15 mL conical tube for every half of a 6-well plate.

***Note:*** As mentioned earlier, ensure that only organoids with sufficient space in BME and a diameter of 150–250 μm are collected at this step. Organoids of appropriate size are essential for achieving successful differentiation.

75. Add ice-cold AdDF+++ up to 12 mL and centrifuge at 200 x g for 3 min at 4°C. Carefully remove as much supernatant as possible, including the translucent BME layer. Repeat this step twice to ensure thorough washing.

**CRITICAL:** Complete removal of residual growth factors and remaining BME is essential for successful differentiation. Any leftover factors from the expansion phase can interfere with differentiation efficiency. Therefore, it is crucial to remove the supernatant as thoroughly as possible, leaving only the organoid pellet, to optimize differentiation outcome.

76. Replate the collected organoids using the same total volume of BME as in the original culture. For example, if a full 6-well plate of organoids was collected, the total replating volume should be 1.2 mL. To achieve this:
  a. Resuspend the organoid pellet evenly in 200 μL of differentiation medium.
  b. Add 1 mL of BME and gently mix to ensure uniform distribution, avoiding bubble formation.
  c. Plate the BME-organoid mixture as droplets of approximately 10 μL each. This should yield approximately 20 droplets per well in the case of a 6-well plate.

**CRITICAL:** Since the organoids are large, they may settle at the bottom of the BME-organoid mixture during plating. To ensure an even distribution of organoids across droplets, stir the suspension with the pipette tip while inside the mixture and pipette up and down a few times before transferring the BME-organoid mixture from the tube into the plate. This prevents organoids from accumulating at the bottom and ensures uniform plating.

77. Carefully invert the plate and leave it at 22°C for approximately 3 min.
78. Transfer the plate to a 37°C, 5% CO_2_ incubator and incubate for 30 min to BME solidify.
79. After incubation, gently add 2 mL of differentiation medium (DM) per well by carefully dispensing along the inner well of the plate to avoid disturbing the BME droplets. Transfer the plate to a 37°C, 5% CO_2_ incubator.
80. The following day, replace the medium with fresh DM. Continue refreshing the medium every 2–3 days while monitoring changes in organoid morphology.
81. At 3 to 5 days after switching to DM, examine the organoid morphology under a microscope to assess whether they have transitioned from “bunch-of-grapes” shape (Figure 6D) to a “sphere-with-lumen” shape (Figure 6E). Unlike the dense, grape-like structures observed in expansion medium, organoids at this stage should appear cystic and no longer proliferative, indicating a transition toward differentiation. Once this morphological change is evident, the next differentiation step can be initiated (see problem 4).

**CRITICAL:** While organoids typically transition to a cystic shape within 3 to 5 days, this process may take longer in some cases. If necessary next step can be delayed for up to 8 days until the organoids exhibit a clear cystic morphology. As long as the organoids appear healthy and show no signs of cell death (Figure 6F), it is important to wait until all organoids have fully transitioned to the cystic “sphere-with-lumen” shape in DM before proceeding.

82. Once the organoids have acquired a cystic morphology, replace the DM with DM+RR to proceed with the next phase of differentiation.
83. Refresh the DM+RR every 2–3 days with fresh medium and incubate for a total of 5 days to allow further differentiation.

***Note:*** At this stage, no further significant morphological changes are observed. By day 5 of DM+RR differentiation, the expression of functional cTEC and mTEC markers (e.g., *Aire, Fezf2, H2-Aa, Psmb11, Cxcl12*) can be confirmed via flow cytometry or qPCR. However, organoids can be maintained in DM+RR for at least 10 days, as long as they remain viable.

***Note:*** During culture in DM+RR, cell debris may be observed floating in the medium. This is normal and will be removed during medium refreshment.

### Organoid characterization and analysis

**Timing: 1 h (for RNA isolation, steps 79–86)**

**Timing: 1.5–2.0 h (for flow cytometry, including antibody incubation, step 87a)**

This step describes methods for characterizing TEC organoids through RNA isolation and flow cytometry analysis. RNA isolation enables downstream applications such as cDNA synthesis and qPCR, allowing for the assessment of gene expression profiles in differentiated cTECs and mTECs. Additionally, flow cytometry provides a means to analyze the cellular composition and protein expression of TEC organoids. These techniques enable researchers to validate organoid formation and differentiation, as well as investigate the functional characteristics relevant to TEC biology.

84. For RNA isolation, collect the organoids following the same method used for replating and passaging (see step 23a–d). Organoids cultured in expansion medium and those differentiated in DM+RR should be collected using the same procedure.
85. Add ice-cold PBS up to 12 mL, then invert the tubes several times to mix thoroughly.
86. Centrifuge at 500 x g for 5 min at 4°C.
87. Carefully remove all supernatant, including the slightly translucent BME layer, leaving only the organoid pellet at the bottom.
88. Resuspend the pellet in 1 mL of ice-cold PBS, then add 10 mL of additional ice-cold PBS and invert the tube to mix thoroughly.
89. Centrifuge at 500 x g for 5 min at 4°C, then carefully remove all supernatant.
90. Use the organoid pellet directly for RNA isolation. Any commercially available RNA isolation kit can be used.

***Note:*** Thorough removal of BME, leaving only the organoid pellet, is crucial for efficient RNA isolation.

***Note:*** The isolated RNA can be analyzed using standard laboratory techniques, including cDNA synthesis followed by qPCR or RNA sequencing.

91. For flow cytometry, collect the organoids following the same method as in RNA isolation (see also step 23a–d). Organoids cultured in expansion medium and in DM+RR should be collected using the same procedure.
92. Add ice-cold AdDF+++ up to 12 mL, then invert the tube several times to mix thoroughly.
93. Centrifuge at 500 x g for 5 min at 4°C, then carefully remove all supernatant, including the slightly translucent BME layer, as much as possible.
94. Add 2 mL of TrypLE, resuspend thoroughly, and incubate in a 37°C water bath for 5 min. Gently swirl the tube every minute to prevent organoids from settling at the bottom.
95. Using a 1000 μL pipette tip, vigorously resuspend the organoids until single cell (see also step 41).
  a. Attach a 20 μL pipette tip to the 1000 μL tip.
  b. Pipette up and down until the medium becomes cloudy, indicating successful dissociation.
96. Pass the cell suspension through a 40 μm cell strainer to remove larger cell clumps that have not fully dissociated into single cells.

***Note:*** TrypLE digestion is known to not significantly cleave surface-expressed proteins, ensuring that antibody staining efficiency remains unaffected during short-term digestion. However, to minimize any potential impact on flow cytometry analysis, limit the TrypLE incubation time to 5 min. Instead, vigorous pipetting using a P1000 with a 20 μL tip attached to the 1000 μL tip is employed to maximize single-cell dissociation while preserving surface marker.

97. Add 10 mL of 1% FBS-containing PBS (FACS buffer) and mix thoroughly. Centrifuge at 500 x g for 5 min at 4°C. Carefully remove the supernatant, leaving only the pellet.
98. Resuspend the pellet in 1 mL of FACS buffer, then add an additional 10 mL of FACS buffer and mix well. Centrifuge again under the same conditions, then carefully remove all supernatant, leaving only the pellet.
99. Count the cells and adjust the cell concentration so that 1 X 10^6^ cells are suspended in 100 μL of FACS buffer. Use 1 X 10^6^ cells per staining condition.

***Note:*** Single-cell suspensions obtained from organoids cultured in expansion medium or differentiated organoids can be analyzed using standard flow cytometry staining protocols commonly employed in various laboratories. Ensure that the appropriate cell number and antibody volume are used according to the specific antibodies being tested.

100. As an example, the following staining procedure can be used.
  a. Resuspend 1 X 10^6^ cells in 100 μL of FACS buffer.
  b. Add the appropriate amount of fluorophore-conjugated antibodies, including anti-mouse Ly-51-PE-Cy7 (RRID: AB_2566112), anti-mouse I-A/I-E-BV421 (RRID: AB_467689), anti-mouse CD80-BV605 (RRID: AB_11126141), and UEA-1-Rhodamine, at the concentrations recommended by the manufacturer. Ly-51 is a marker for cTEC, while UEA-1 labels mTEC. I-A/I-E and CD80 are used to evaluate functional maturation of TECs.
  c. Incubate the samples on ice for 30 min in the dark to allow proper staining.
  d. Wash the cells by adding 10 mL of FACS buffer, then centrifuge at 500 x g for 5 min at 4°C and discard the supernatant, leaving only the pellet.
  e. Resuspend stained cells with 500 μL of FACS buffer for flow cytometry analysis.

### Additional applications

***Note:*** In addition to qPCR and flow cytometry, TEC organoids can be further analyzed using immunofluorescence staining^5^ and genetically modified through CRISPR-Cas9-mediated gene editing^6^^,^^7^ or electroporation-based techniques.^8^ These methods have been previously established for organoid systems and can be applied to TEC organoids without the need for adjustments by following published protocols. Researchers are encouraged to refer to existing studies for specific methodologies and experimental optimizations.

## Expected outcomes

This protocol enables the efficient generation and long-term expansion of 3D mouse TEC organoids. When cultured in expansion medium, organoids are typically visible within 2–4 days as small, dense structures, marking the initial formation from adult mouse thymus tissue. These organoids continue to grow and can be passaged approximately every 10–14 days, allowing for extended culture and experimental applications.

Upon transitioning to differentiation conditions (DM, DM+RR), TEC organoids undergo morphological changes, adopting a cystic shape within 3–5 days in DM, with some cases taking up to 8 days. By day 5 in DM+RR, TEC organoids express lineage-specific markers characteristic of cortical and medullary thymic epithelial cells, which can be confirmed using qPCR or flow cytometry.

TEC organoids generated through this protocol are suitable for various downstream applications, including single-cell analysis, transcriptomic profiling, and functional assays such as thymocyte co-culture.^1^^,^^9^ The organoids are also compatible with standard techniques such as immunofluorescence staining, CRISPR-Cas9-mediated gene editing, and electroporation-based genetic engineering, allowing researchers to explore TEC biology in greater depth.

## Limitations

TEC organoids generated using this protocol are cultured under artificial conditions and lack non-epithelial components, such as resident immune cells, blood vessels and stromal cells. Therefore, this system is best suited for studying TEC biology and TEC-thymocyte interactions rather than modeling the full complexity of the thymic microenvironment. Researchers aiming to explore immune cell crosstalk within the thymus may need to incorporate additional co-culture models or *in vivo* validation.

Additionally, the differentiation process described here does not fully recapitulate *in vivo* TEC heterogeneity. While the protocol supports the generation of cTECs and mTECs, their functional properties and ratios may differ from those observed in the native thymus. For instance, MHC class II expression in organoid derived TECs is lower than in primary TECs, which may affect certain functional assays, such as thymocyte selection.^1^ Further optimization of differentiation conditions may be required to enhance the generation of functionally mature TEC subsets.

Finally, while this protocol enables the long-term expansion of TEC organoids, the isolation method has not been optimized for the selective enrichment of thymic epithelial progenitors. As a result, the presence and contribution of thymic epithelial stem cells (TESCs) within the culture remain undefined. Future protocol modifications, such as optimizing tissue processing, incorporating specific population sorting, and refining media composition with selective growth factors, may help characterize and utilize TESCs more effectively.

## Troubleshooting

### Problem 1

Poor organoid formation after initial plating from tissue processing.

### Potential solution

If organoid formation is inefficient in the initial plating from tissue processing, this may be due to incomplete mechanical dissociation, leading to excessive tissue clumps remaining on the cell strainer. This can reduce the number of viable epithelial fragments available for seeding. To improve dissociation, ensure that tissue fragments are sufficiently broken down by pipetting up and down thoroughly during the straining process. If large aggregates persist, additional gentle mechanical dissociation (e.g., repeated pipetting with a flame-narrowed Pasteur pipette) may be necessary.

Additionally, check the BME concentration and handling temperature, as incomplete polymerization or premature solidification can impact plating efficiency. When necessary, optimize seeding density by performing gradient seeding (i.e., testing different cell densities per droplet) to determine the optimal conditions for TEC organoid formation.

### Problem 2

Loss of TEC organoids during re-plating or passaging due to inefficient separation from BME.

### Potential solution

BME can form gel-like residues that trap organoids, leading to inefficient recovery during both re-plating and passaging. This issue is particularly pronounced during re-plating steps, as TrypLE is not used, increasing the risk of organoid loss. To minimize this, pre-treat pipette tips and conical tubes with cold AdDF+++ to reduce adhesion. Additionally, ensure that BME is sufficiently disrupted by thorough pipetting before transferring organoids. When re-plating organoids for differentiation, dilution, or after initial plating, use gentle but consistent pipetting to prevent clumping and ensure uniform redistribution. If organoids remain embedded in residual BME, consider an additional brief wash step using ice-cold AdDF+++ before centrifugation to improve recovery.

### Problem 3

TEC organoids appear dense and fail to develop a grape-like morphology in the expansion medium.

### Potential solution

Overcrowding of organoids within the BME droplets can lead to limited space for expansion, resulting in compact and non-distinct structures. Additionally, when organoids are densely packed, those located at the center of the droplet may experience reduced nutrient and oxygen diffusion, leading to lower viability.

To resolve this, adjust the passaging ratio to provide sufficient room for expansion. Instead of standard passaging, consider higher dilution plating, such as redistributing organoids from a single well of a 6-well plate into ten wells of new 6-well plates or plating only a fraction of the collected cells into fresh BME. This ensures that organoids have adequate space for proper growth, reduces overcrowding, and prevents viability loss in organoids trapped within the dense droplet core.

By implementing these adjustments, TEC organoids can expand efficiently and acquire the characteristic grape-like morphology, ensuring a well-structured 3D architecture suitable for downstream applications.

### Problem 4

TEC organoids fail to adopt a “sphere-with-lumen” morphology during differentiation, in DM.

### Potential solution

TEC organoids typically transition into a “sphere-with-lumen” structure within 5 days in DM, though some may take up to 8 days. If this process is delayed or incomplete, ensure that organoids are not too small at the time of differentiation, as smaller structures may lack sufficient cellular mass to be differentiated.

Additionally, differentiation medium (DM) is formulated by removing specific components from the expansion medium (EM), rather than by adding new differentiation factors. As a result, any residual EM components may interfere with differentiation efficiency. To prevent this, perform thorough washing steps to remove residual EM and BME before switching to DM (Step 74 and 75). This includes: (1) Gentle but thorough pipetting during washes to minimize organoid loss while ensuring complete disruption and removal of residual BME and EM components. (2) Centrifugation to separate organoids from the wash solution. (3) Carefully remove the supernatant to ensure residual BME and EM are fully removed.

If organoids fail to adopt a cystic morphology after these adjustments, do not proceed to the next differentiation stage (DM+RR). Instead, restart differentiation from EM, ensuring that all washing and seeding conditions are optimized before differentiation.

### Problem 5

TEC organoids attach to the plate surface and grow in 2D rather than forming 3D structures.

### Potential solution

Occasionally, TEC organoids may adhere to the bottom of the plate and start growing as a 2D monolayer, which can compromise their structural and functional integrity. This issue often arises due to insufficient BME coverage, improper droplet formation, or BME spreading upon plating.

To prevent 2D attachment, ensure that: (1) BME droplets are sufficiently thick to fully encapsulate the organoids and prevent direct contact with the plate. (2) Plating is done carefully by gently pipetting the BME-cell (or -organoid) mixture to form rounded droplets without spreading. (3) Plates are immediately inverted after BME has been plated to reduce the chance of organoids settling on the bottom.

If organoids have attached to the plate and started growing in 2D, do not attempt to recover them. Instead, carefully collect only the unaffected 3D organoids, resuspend them in fresh BME, and re-plate them to maintain proper 3D culture conditions.

### Problem 6

TEC organoids exhibit poor survival after freezing and thawing.

### Potential solution

TEC organoid viability after freezing and thawing can be compromised due to suboptimal cryopreservation techniques, excessive ice crystal formation, or prolonged exposure to DMSO during the thawing process. To improve survival rates, commercially available freezing media optimized for organoid preservation can be used such as Cell Banker I. These reagents help minimize cellular stress and improve post-thaw recovery. Before freezing, ensure that organoid pellets are properly prepared by removing as much residual culture medium as possible. Excess residual medium can lead to unintended dilution of the freezing medium, which may reduce cryoprotective efficiency. After centrifugation, carefully discard the supernatant without disturbing the pellet, and resuspend the organoids directly in the appropriate freezing medium. Additionally, during thawing, rapidly dilute the cryopreservation medium with ice-cold AdDF+++ to minimize exposure to cryoprotective agents (e.g., DMSO). After thawing, passage the organoids at least once before experimental use to allow recovery and stabilization.

## Resource availability

### Lead contact

Further information and requests for resources and reagents should be directed to and will be fulfilled by the lead contact, Sangho Lim (s.lim@hubrecht.eu).

### Technical contact

For technical questions regarding the execution of this protocol, please contact Sam Willemsen (s.willemsen@hubrecht.eu).

### Materials availability

This study did not generate new unique reagents. All commercially available reagents and materials used in this protocol, including organoid culture media and supplements, are listed in the key resources table**.** Researchers interested in obtaining specific components should refer to their respective suppliers.

### Data and code availability

This study does not include original code or datasets.

## Acknowledgments

This work was supported by the Netherlands Organ-on-Chip Initiative, a NOW Gravitation project (024.003.001) funded by the Ministry of Education, Culture, and Science of the government of the Netherlands (H.C. and S.L.).

## Author contributions

S.L. and S.W. conducted the experiments, drafted the manuscript, and contributed to writing. N.L.W.E.Y. performed the experiments. J.H.v.E. coordinated animal experiments and reviewed and proofread the manuscript. H.C. reviewed and proofread the manuscript.

## Declaration of interests

H.C. is the head of Pharma Research and Early Development at Roche, Basel, and holds several patents related to organoid technology. The full disclosure is given at http://www.uu.nl/staff/JCClevers/.

## References

[1] Lim S., J F van Son G., Wisma Eka Yanti N.L., Andersson-Rolf A., Willemsen S., Korving J., Lee H.G., Begthel H., Clevers H. (2024). Derivation of functional thymic epithelial organoid lines from adult murine thymus. Cell Rep..

[2] Abramson J., Anderson G. (2017). Thymic Epithelial Cells. Annu. Rev. Immunol..

[3] Alves N.L., Huntington N.D., Rodewald H.R., Di Santo J.P. (2009). Thymic epithelial cells: the multi-tasking framework of the T cell “cradle”. Trends Immunol..

[4] Xing Y., Hogquist K.A. (2014). Isolation, identification, and purification of murine thymic epithelial cells. J. Vis. Exp..

[5] Dekkers J.F., Alieva M., Wellens L.M., Ariese H.C.R., Jamieson P.R., Vonk A.M., Amatngalim G.D., Hu H., Oost K.C., Snippert H.J.G. (2019). High-resolution 3D imaging of fixed and cleared organoids. Nat. Protoc..

[6] Celotti M., Derks L.L.M., van Es J., van Boxtel R., Clevers H., Geurts M.H. (2024). Protocol to create isogenic disease models from adult stem cell-derived organoids using next-generation CRISPR tools. STAR Protoc..

[7] Artegiani B., Hendriks D., Beumer J., Kok R., Zheng X., Joore I., Chuva de Sousa Lopes S., van Zon J., Tans S., Clevers H. (2020). Fast and efficient generation of knock-in human organoids using homology-independent CRISPR-Cas9 precision genome editing. Nat. Cell Biol..

[8] Fujii M., Matano M., Nanki K., Sato T. (2015). Efficient genetic engineering of human intestinal organoids using electroporation. Nat. Protoc..

[9] Montel-Hagen A., Sun V., Casero D., Tsai S., Zampieri A., Jackson N., Li S., Lopez S., Zhu Y., Chick B. (2020). In Vitro Recapitulation of Murine Thymopoiesis from Single Hematopoietic Stem Cells. Cell Rep..

